# PANI Branches onto Donor-Acceptor Copolymers: Synthesis, Characterization and Electroluminescent Properties of New 2D-Materials

**DOI:** 10.3390/polym10050553

**Published:** 2018-05-21

**Authors:** Ignacio A. Jessop, Fernando R. Díaz, Claudio A. Terraza, Alain Tundidor-Camba, Ángel Leiva, Linda Cattin, Jean-Christian Bèrnede

**Affiliations:** 1 Departamento de Química, Facultad de Ciencias, Universidad de Tarapacá, P.O. Box 7-D, Av. General Velásquez 1775, Arica, Chile; 2 Departamento de Química Orgánica, Facultad de Química, Pontificia Universidad Católica de Chile, P.O. Box 306, Post 22, Santiago, Chile; fdiaz@uc.cl (F.R.D.); cterraza@uc.cl (C.A.T.); atundido@uc.cl (A.T.-C.); 3 UC Energy Research Center, Pontificia Universidad Católica de Chile, P.O. Box 306, Post 22, Santiago, Chile; 4 Departamento de Química Física, Facultad de Química, Pontificia Universidad Católica de Chile, P.O. Box 306, Post 22, Santiago, Chile; aleivac@uc.cl; 5 Institut des Matériaux Jean Rouxel (IMN), CNRS, UMR 6502, Université de Nantes, 2 rue de la Houssinière, BP 32229, 44322 Nantes CEDEX 3, France; Linda.Cattin-Guenadez@cnrs-imn.fr; 6 MOLTECH-Anjou, CNRS, UMR 6200, Université de Nantes, 2 rue de la Houssinière, BP 92208, F-44000 Nantes, France; Jean-Christian.Bernede@univ-nantes.fr

**Keywords:** donor-acceptor copolymer, polyaniline, 2D-conjugated polymers, OLEDs

## Abstract

A new series of two-dimensional statistical conjugated polymers based on aniline and 9,9-dihexylfluorene as donor units and benzo- or naphtho-quinoxaline/thiadiazole derivatives as acceptor moieties, possessing PANI segments as side chains, were designed and synthesized. To investigate the effects of the perpendicular PANI branches on the properties of the main chain, the optical, electrochemical, morphological and electroluminescence properties were studied. The 2D materials tend to possess lower molecular weights and to absorb and to emit light red-shifted compared to the trunk 1D-polymers, in the yellow-red region of the visible spectrum. The 1D- and 2D-conjugated polymers present optical band gaps ranging from 2.15–2.55 eV, HOMO energy levels between −5.37 and −5.60 eV and LUMO energy levels between −3.02 and −3.29 eV. OLED devices based on these copolymers were fabricated. Although the performances were far from optimal due to the high turn-on voltages for which electroluminescence phenomena occur, a maximum luminescence of 55,100 cd/m^2^ together with a current density of 65 mA/cm^2^ at 18.5 V were recorded for a 2D-copolymer, **PAFC6TBQ-PANI**.

## 1. Introduction

Tremendous progress has been achieved in the development of conducting polymers (CPs) over the last couple of decades, due to their potential applications in optoelectronics fields such as organic solar cells (OSCs) [1,2,3,4], organic field-effect transistors (OFETs) [5,6,7] and organic light-emitting diodes (OLEDs) [8,9,10,11], among others. Since each of these fields requires CPs with a specific set of both physical and electrochemical features, several structural modification approaches have been used to modulate their properties. Among them, alternate donor-acceptor monomers in the conjugated polymer backbone (D-A CPs) allow efficient tuning of their electronic properties by intramolecular charge transfer (ICT) [12,13,14,15,16].

Several studies have described the excellent electrochromic, photovoltaic and electroluminescent features of D-A CPs based on fluorene and/or triarylaniline as donor units; thereby, they have been representative benchmark materials in OLEDs and OSCs [17,18,19,20]. However, to the best of our knowledge, there are few published works about D-A CPs containing aniline and fluorene together as donor moieties [21,22]. CPs with free amino groups would allow their subsequent functionalization, tuning their optical and electronic properties by covalently attaching azo dyes or imine-chromophores or growing polyaniline (PANI) chains onto their backbones, generating a two-dimension (2D)-conjugated polymer. 2D-CPs have demonstrated several interesting features, such as high hole-transporting properties, isotropic charge transport and ICT, and have shown better photovoltaic performances in comparison with the polymers without conjugated side chains [16,23,24,25,26].

Therefore, in this article, we report the synthesis and characterization of statistical D-A CPs (1D-CPs) derived from 2,6-dibromoaniline with the pinacol boronate ester of 9,9-dihexylfluorene as donor units and benzo- or naphto-quinoxaline/thiadiazole derivatives as acceptor moieties. While the fluorene units and free amino groups would help to improve the solubility of the polymers through their attached alkyl chains, acceptor moieties would allow tuning the optical and electronic properties of the resulting materials through promoting the ICT effect. The prepared 1D- and 2D-CPs were characterized by NMR, UV-Vis and fluorescence spectroscopies. Furthermore, size-exclusion chromatography (SEC), cyclic voltammetry (CV) and scanning-electron microscopy (SEM) were measured. Furthermore, OLED devices were fabricated and tested using the CPs as emissive layers. We expected that the incorporation of PANI segments would allow modulating the HOMO energy level of 1D-CPs, through the extension of conjugation at the direction perpendicular to the polymer backbone, as well as acting as a hole-transport layer (HTL), providing dual HTL/electroluminescent (EL) materials [27,28]. Besides, due to the presence of benzo- or naphtho-quinoxaline/thiadiazole derivatives, the materials should emit light between the yellow-red regions of the visible spectrum [29,30].

The functionalization of 1D-CPs produced new 2D-materials with irregular segments of PANI as side-chains, but with lower molecular weights. Partial overlapping of the p-orbitals of the 1D-CPs with the p-orbitals of PANI segments was observed in the UV-Vis and fluorescence spectra. As was expected, the polymers emitted light in the range of the yellow-red region. 1D-CPs and 2D-CPs exhibited optical bandgaps ranging from 2.15 eV to 2.55 eV and HOMO and LUMO energy levels properly aligned with the HOMO of MoO_3_ and with the LUMO of C_60_, the hole-transport and the electron-injector layers, respectively, used in the manufacture of OLEDs. Besides, most of the materials form good films, with smooth and homogeneous surfaces. Preliminary results of the OLED devices based on the polymers as emissive layers showed low performances, with high values of the turn-on voltages (>13 V). The best results were obtained with the **PAFC6TBQ-PANI**-based device, which presented a maximum luminescence of 55,100 cd/m^2^ and a current density of 65 mA/cm^2^. However, after reaching this value, the device became irretrievably ruined. This study provides a starting point for a new class of 2D-materials and motivates future researches regarding the modification or improvement of the chemical structures of 2D-CPs, to produce OLED devices based on them with better performances.

## 2. Materials and Methods

### 2.1. Materials

All reagents and solvents were used without further purification. 2,6-Dibromoaniline (**AniBr**), 9,9-dihexylfluorene-2,7-diboronic acid bis(1,3-propanediol) ester (**FC6B**), 4,7-dibromobenzo[*c*]-1,2,5-thiadiazole (**BTDzBr**), 2,2′-thenil, tetrakis(triphenylphosphine)palladium(0) (Pd[PPh_3_]_4_), iron(III) chloride anhydrous (FeCl_3_), hydrazine monohydrate (N_2_H_6_∙H_2_O), aliquat® 336, phenylboronic acid, bromobenzene and tetrabutylammonium hexafluorophosphate (TBAHFP) were obtained from Aldrich Chemical (Milwaukee, WI, USA). Thionyl chloride (SOCl_2_), pyridine (Py), sodium borohydride (NaBH_4_), potassium carbonate (K_2_CO_3_) and the other reagents and solvents were purchased from Merck Millipore (Darmstadt, Germany). Compounds 3,6-dibromo-1,2-phenylenediamine (**DABBr**), 1,4-dibromo-2,3-naphthalenediamine (**DANBr**), 4,9-dibromonaphtho[2,3-*c*][1,2,5]thiadiazole (**NTDzBr**), 5,8-dibromo-2,3-di(thien-2-yl)quinoxaline (**TQBr**) and 5,10-dibromo-2,3-di(thien-2-yl)benzo[*g*]quinoxaline (**TBQBr**) were prepared according to previously described methods [31,32,33].

### 2.2. Measurements

^1^H and ^13^C NMR spectra were obtained from a Bruker AVANCE III HD 400 spectrometer (Billerica, MA, USA) Chemical shifts were reported as δ values (ppm) relative to an internal tetramethylsilane (TMS) standard. FTIR spectra were run on a Bruker Vector 22 spectrophotometer (Bruker Optik GmbH, Bremen, Germany) over the range of 450–4000 cm^−1^. Number-average (*M_n_*) and weight-average (*M_w_*) molecular weights were determined by size exclusion chromatography (SEC) at 25 °C with a high-pressure liquid chromatography (HPLC) Knauer pump, three PLgel 5 μMixed-C columns and astatic light-scattering (EA-02 Dawn Eos Enhanced Optical System) detector, using tetrahydrofuran (THF) as the eluent. The calibration curve was made with a series of monodisperse polystyrene standards. UV-Vis absorption spectra were recorded with an Analitik Jena SPECORD 40 spectrophotometer (Jena, Germany) using 1-cm path length quartz cells. Fluorescence spectra were recorded using a LS55 PerkinElmer fluorescence spectrometer (Perkin-Elmer, Waltham, MA, USA). The samples were excited at the wavelength of maximum absorption of the polymers in solution. For solid-state measurements, polymer solutions (10 mg/mL in chloroform) were cast on fluorine-doped tin oxide (FTO) glass plates (Sigma Aldrich, Milwaukee, WI, USA). Cyclic voltammograms (CV) were taken with a Voltamaster BAS CV-50W potentiostat/galvanostat (Radiometer Analytical SAS, Villeurbanne CEDEX, France) at a scan rate of 100 mV/s using polymer-coated slides as working electrodes, Ag/AgCl (in tetramethylammonium chloride solution) as the reference electrode and a Pt coil as the counter electrode in an anhydrous and argon-saturated solution of 0.1 M of tetrabutylammonium hexafluorophosphate (Bu_4_NPF_6_) in acetonitrile. The Ag/AgCl working electrode response was adjusted to match with the potential of saturated calomel electrode (SCE) response at 20 °C [34]. In these conditions, the oxidation onset potential (*E_on_^ox^*) of ferrocene was 0.40 V versus SCE.

### 2.3. Electroluminescent Device Fabrication and Characterization

The OLED devices were fabricated on an indium-tin oxide (ITO) coated glass substrate (Solems, Palaiseau, France) (10 mm × 25 mm with a sheet resistance of about 25 Ω/square). The ITO-coated glass was partially etched with zinc powder and hydrochloric acid and then scrubbed with soap, rinsed with deionized water, dried with an air flow and placed into a vacuum chamber (10^−4^ Pa) [35]. For the EL measurements, the organic multilayer planar heterojunction (PHJ) devices’ configurations were glass/ITO/MoO_3_ (3 nm)/polymer (50–60 nm)/C_60_ (40 nm)/bathocuproine (BCP) (10 nm)/Al (100 nm), where MoO_3_ was used as a hole-injecting material (HIL) and BCP was used as the hole-blocking material (HBL). The MoO_3_, C_60_, BCP and aluminum layers were placed onto the substrate by sublimation. The thin-film deposition rates (0.05–0.15 nm/s) and thicknesses (3–100 nm) were measured in situ using a quartz monitor. The polymer layers were deposited onto the substrates (glass/ITO/MoO_3_) by dip coating. Then, 0.1 M solutions of polymers in chloroform were prepared, and 1–6 coating cycles were applied to the substrate. The substrate was immersed at an angle orthogonal to the liquid surface, at a constant speed. The thickness of the films deposited by dip coating was estimated by cross-section visualization, using a JEOL 7600F scanning electron microscope (SEM) (JEOL, Tokyo, Japan), and it was determined to be around 15 nm per cycle. Observation of the cross-section and the surface morphology of the layers was performed using a JEOL 6400F field-effect SEM (JEOL, Tokyo, Japan). The EL devices were characterized by current-voltage (I-V) and electroluminescence-voltage (EL-V) measurements. The I-V characteristics were recorded by a Keithley 617 programmable electrometer (Keithley Instruments Inc., Cleveland, OH, USA), a Keithley 2000 multimeter (Keithley Instruments Inc., Cleveland, OH, USA) and a Lambda IEEE-488 programmable power supply Model LLS6060-GPIP (TDK-Lambda Corporation, Hong Kong, China) interfaced with an IBM-PC computer (Armonk, NY, USA). The EL-V characteristics were recorded through the transparent ITO electrode and glass. The light output was detected using a silicon photodiode and a Keithley 617 electrometer. The active area of the devices in this study was 8 mm^2^. All device characterizations were carried out in the ambient atmosphere.

### 2.4. Polymer Synthesis

General procedure for the synthesis of 1D-CPs: A mixture composed of **TQBr**, **TBQBr**, **BTDzBr** or **NTDzBr** (1.00 mmol), **AniBr** (1.00 mmol), **FC6B** (2.00 mmol), Pd[PPh_3_]_4_ (0.06 mmol), Aliquat® 336 (0.6 mmol) and K_2_CO_3_ (48 mmol) was purged under a steady stream of N_2_ for 30 min at room temperature. Degassed toluene (12.5 mL) and degassed water (7.5 mL) were then added to the reaction mixture. After heating at 90 °C for 48 h under the N_2_ atmosphere, the reaction mixture was treated with phenylboronic acid. After additional heating for 2 h, bromobenzene was added, and the reaction mixture was heated for 2 h more to complete the end-capping process. After cooling at room temperature, the reaction mixture was poured into methanol (200 mL), and the resulting solid was filtered through a Soxhlet thimble. The solid was extracted with acetone, *n*-hexane and finally with chloroform until the wash solution of each extraction was colorless. The solvent was removed from the chloroform extract, and the product was recrystallized from methanol, collected by filtration and dried under vacuum to give **PAFC6TQ**, **PAFC6TBQ**, **PAFC6BTDz** or **PAFC6NTDz**, respectively.

**PAFC6TQ:** 0.69 g of a brown-yellow solid (yield 40%). ^1^H NMR (CDCl_3_, 400 MHz): δ (ppm) 8.02–6.61 (m, arom.), 4.28–3.77 (m, N-H), 2.35–1.81 (m, aliph.), 1.28–0.48 (aliph.). FTIR (KBr, cm^−1^): 3474 (ν N-H amino groups), 3385 (ν N-H amino groups), 3054 (ν C-H arom. rings), 3028 (ν C-H arom. rings), 2951 (ν C-H alkyl chains), 2925 (ν C-H alkyl chains), 2853 (ν C-H alkyl chains), 1607 (ν C=C arom. rings), 1560 (ν C=N quinoxaline rings), 1454 (ν C=C arom. rings), 850 (γ C-H 2-subst. thiophenes), 703 (γ C-H 2-subst. thiophenes).

**PAFC6TBQ:** 0.42 g of an orange solid (yield 24%). ^1^H NMR (CDCl_3_, 400 MHz): δ (ppm) 8.23–6.67 (m, arom.), 4.36–3.57 (m, N-H), 2.24–1.79 (m, aliph.), 1.35–0.56 (m, aliph.). FTIR (KBr, cm^−1^): 3482 (ν N-H amino groups), 3385 (ν N-H amino groups), 3067 (ν C-H arom. rings), 2951 (ν C-H alkyl chains), 2925 (ν C-H alkyl chains), 2852 (ν C-H alkyl chains), 1607 (ν C=C arom. rings), 1560 (ν C=N quinoxaline rings), 1452 (ν C=C arom. rings), 850 (γ C-H 2-subst. thiophenes), 704 (γ C-H 2-subst. thiophenes).

**PAFC6BTDz:** 0.42 g of a brown-yellow solid (yield 27%). ^1^H NMR (CDCl_3_, 400 MHz): δ (ppm) 8.19–6.60 (m, arom.), 4.29–3.61 (m, -NH_2_), 2.31–1.82 (m, aliph.), 1.29–0.45 (m, aliph.). FTIR (KBr, cm^−1^): 3455 (ν N-H amino groups), 3386 (ν N-H amino groups), 3058 (ν C-H arom. rings), 2951 (ν C-H alkyl chains), 2925 (ν C-H alkyl chains), 2852 (ν C-H alkyl chains), 1606 (ν C=C arom. rings), 1543 (ν C=N thiadiazole rings), 1450 (ν C=C arom. rings).

**PAFC6NTDz:** 0.67 g of a red solid (yield 42%). ^1^H NMR (CDCl_3_, 400 MHz): δ (ppm) 8.22–6.74 (m, arom.), 4.30–3.57 (m, -NH_2_), 2.29–1.63 (m, aliph.), 1.41–0.32 (m, aliph.). FTIR (KBr, cm^−1^): 3448 (ν N-H amino groups), 3386 (ν N-H amino groups), 3057 (ν C-H arom. rings), 3033 (ν C-H arom. rings), 2952 (ν C-H alkyl chains), 2925 (ν C-H alkyl chains), 2853 (ν C-H alkyl chains), 1608 (ν C=C arom. rings), 1544 (ν C=N thiadiazole rings), 1461 (ν C=C arom. rings).

General procedure for the synthesis of 2D-CPs: A mixture composed of **PAFC6TQ**, **PAFC6TBQ**, **PAFC6BTDz** or **PAFC6NTDz** (0.40 mmol), aniline (2.00 mmol), anhydrous FeCl_3_ (5.00 mmol) and CHCl_3_ was stirred under an N_2_ atmosphere at 0–5 °C for 24 h in the dark. After evaporation of the solvent, 100 mL of a 9:1 (vol:vol) MeOH/H_2_O mixture were added, and the reaction mixture was stirred for 24 h at room temperature. The resultant solid was filtered and dedoped by stirring with 100 mL of a 50% aqueous hydrazine solution for 24 h at room temperature. The obtained solid was then filtered through a Soxhlet thimble and extracted with chloroform until the wash solution was colorless. The solvent was removed from chloroform extract, and the product was recrystallized with methanol, collected by filtration and dried under vacuum to afford **PAFC6TQ-PANI**, **PAFC6TBQ-PANI**, **PAFC6BTDz-PANI** or **PAFC6NTDz-PANI**, respectively.

**PAFC6TQ-PANI:** 0.23 g of a dark-green solid (yield 63%). ^1^H NMR (CDCl_3_, 400 MHz): δ (ppm) 8.13–6.98 (m, arom.), 2.28–1.98 (m, aliph.), 1.34–0.60 (m, aliph.). FTIR (KBr, cm^−1^): 3454 (ν N-H amino groups), 3386 (ν N-H amino groups), 3058 (ν C-H arom. rings), 2951 (ν C-H alkyl chains), 2926 (ν C-H alkyl chains), 2854 (ν C-H alkyl chains), 1607 (ν C=C arom. rings), 1561 (ν C=N quinoxaline rings), 1499 (ν C=C arom. rings), 1453 (ν C=C arom. rings), 850 (γ C-H 2-subst. thiophenes), 704 (γ C-H 2-subst. thiophenes).

**PAFC6TBQ-PANI:** 0.12 g of a brown solid (yield 33%). ^1^H NMR (CDCl_3_, 400 MHz): δ (ppm) 8.15–6.81 (m, arom.), 2.25–1.79 (m, aliph.), 1.29–0.52 (m, aliph.). FTIR (KBr, cm^−1^): 3482 (ν N-H amino groups), 3386 (ν N-H amino groups), 3058 (ν C-H arom. rings), 2951 (ν C-H alkyl chains), 2926 (ν C-H alkyl chains), 2854 (ν C-H alkyl chains), 1607 (ν C=C arom. rings), 1561 (ν C=N quinoxaline rings), 1499 (ν C=C arom. rings), 1453 (ν C=C arom. rings), 850 (γ C-H 2-subst. thiophenes), 704 (γ C-H 2-subst. thiophenes).

**PAFC6BTDz-PANI:** 0.12 g of a brown solid (yield 34%). ^1^H NMR (CDCl_3_, 400 MHz): δ (ppm) 8.19–7.01 (m, arom.), 2.23–1.81 (m, aliph.), 1.32–0.50 (m, aliph.). FTIR (KBr, cm^−1^): 3448 (ν N-H amino groups), 3058 (ν C-H arom. rings), 3034 (ν C-H arom. rings), 2952 (ν C-H alkyl chains), 2926 (ν C-H alkyl chains), 2854 (ν C-H alkyl chains), 1608 (ν C=C arom. rings), 1543 (ν C=N thiadiazole rings), 1508 (ν C=C arom. rings), 1462 (ν C=C arom. rings).

**PAFC6NTDz-PANI:** 0.25 g of a dark-red solid (yield 71%). ^1^H NMR (CDCl_3_, 400 MHz): δ (ppm) 8.17–6.98 (m, arom.), 2.13–1.79 (m, aliph.), 1.31–0.49 (m, aliph.). FTIR (KBr, cm^−1^): 3448 (ν N-H amino groups), 3058 (ν C-H arom. rings), 2953 (ν C-H alkyl chains), 2924 (ν C-H alkyl chains), 2851 (ν C-H alkyl chains), 1605 (ν C=C arom. rings), 1542 (ν C=N thiadiazole rings), 1508 (ν C=C arom. rings), 1450 (ν C=C arom. rings).

## 3. Results and Discussion

### 3.1. Synthesis and Characterization of Polymers

**Scheme 1:**
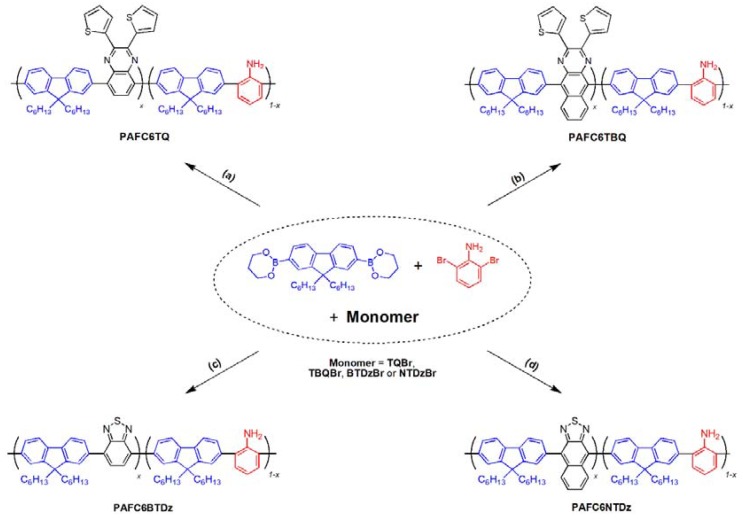
Synthesis of the 1D-conjugated polymers.

As shown in Scheme 1, the 1D-conjugated copolymers were synthesized through a Suzuki polycondensation by using 1.00 eq. of **FC6B**, 0.50 eq. of **AniBr** and 0.50 eq. of the respective acceptor unit. All polymers were fractionated via Soxhlet extraction using acetone, *n*-hexane and chloroform as solvents. The chloroform fraction was recovered, concentrated and the residue precipitated to afford the corresponding polymers. The polymerization yields were rather low (below 50%), even after 48 h, with large amounts of insoluble material remaining in the thimble after Soxhlet extraction. This suggests that the amino groups on aniline units together with the alkyl-chains on the fluorene moieties were not able to facilitate the processability of the high molecular mass fraction on the 1D-CPs. This synthetic pathway has led to highly soluble polymers in common organic solvents, such as chloroform, tetrahydrofuran, chlorobenzene and *o*-dichlorobenzene, with weight-averaged molecular weight (*M_w_*) between 67.8 and 4.8 Kg/mol and polydispersity indexes (PDI) between 2.65 and 1.57 (Table 1).

**Scheme 2:**
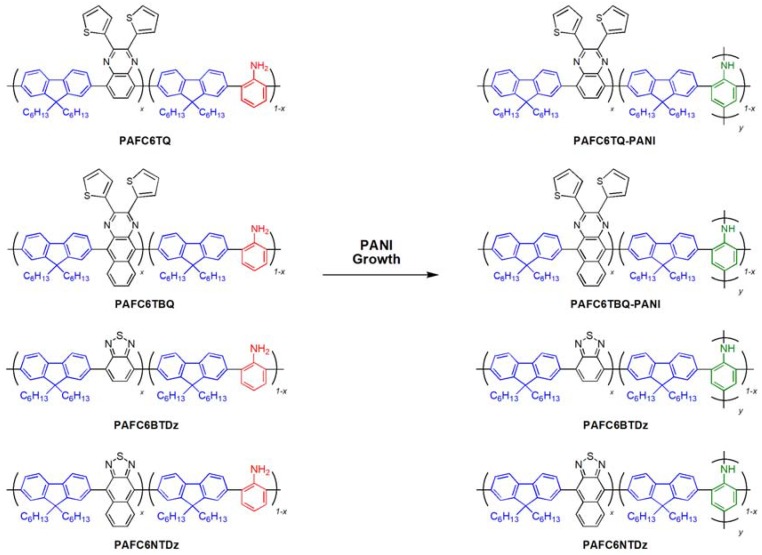
Representation of 2D-conjugated polymer structures.

The in situ growth of PANI onto the statistical D-A CPs was successfully carried out via oxidative polymerization of aniline using FeCl_3_ as the oxidizing agent and chloroform as the solvent (Scheme 2). To ensure that the polymerization reaction occurred on the copolymers’ backbones, an excess of aniline was added to the mixture (5 eq. against 1 eq. of the copolymer). This excess caused PANI and high molecular weight 2D-conjugated polymers to be formed and precipitated during the reaction and could explain the high variability in yields (33–71%) and the lower *M_w_* obtained for the chloroform-soluble 2D-CPs compared with the 1D-CPs (see Table 1).

Chemical structures of the obtained polymers were confirmed by FTIR and NMR techniques. The FTIR spectra of all 1D-CPs exhibited characteristic absorption bands from the N-H stretching vibrations of amino groups between 3485 and 3385 cm^−1^ and from the C-H stretching vibrations of alkyl chains attached to fluorene monomers between 2952 and 2853 cm^−1^. The imino group (C=N) absorptions of quinoxaline and thiadiazole rings were observed at about 1560 and 1540 cm^−1^, respectively [36,37]. Besides, **PAFC6TQ** and **PAFC6TBQ** spectra showed two bands at 850 and 704 cm^−1^, assignable to the C-H out-of-plane vibrations of two-substituted thiophenes [38].

**Figure 1:**
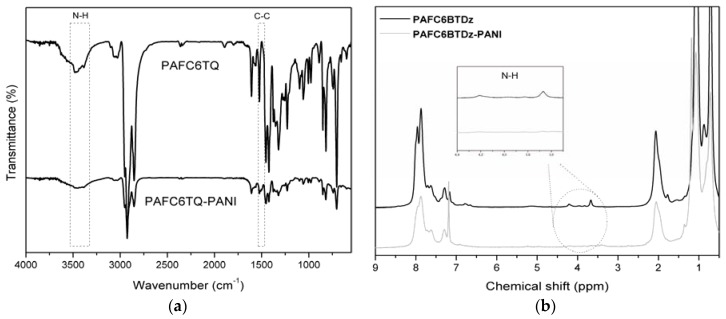
(**a**) FTIR spectra of **PAFC6TQ** and **PAFC6TQ-PANI**; (**b**) ^1^H NMR spectra of **PAFC6BTDz** and **PAFC6BTDz-PANI**.

After PANI growth onto statistical D-A CPs, the N-H stretching bands tends to merge into one broad peak, as can be seen in the example in Figure 1a. Besides, the presence of peaks at 1508 and 1499 cm^−1^ for the thiadiazole- and quinoxaline-based 2D-CPs, respectively, was observed. These signals are due to benzenoid stretching vibrations of repetitive aniline units [39,40]. Both observations are indicative that oligomeric PANI chains or blocks effectively grew or were grafted, respectively, on the 1D-CPs.

Likewise, the almost disappearance of amine protons signal at about 4.0 ppm in the ^1^H NMR spectra of all 2D-CPs provides evidence of some degree of PANI growth onto the statistical D-A CPs (see the example in Figure 1b).

### 3.2. Optical and Electrochemical Properties

**Figure 2:**
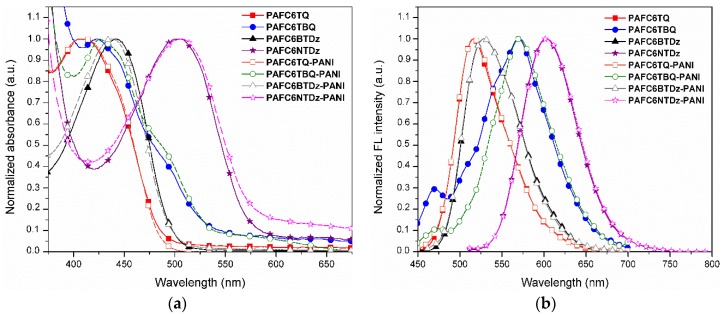
(**a**) Normalized UV-Vis absorption; (**b**) and normalized fluorescence spectra of 1D- and 2D-copolymers as thin films.

The UV-Vis absorption and fluorescence spectra of polymer deposits on FTO glass plates are shown in Figure 2, and the data are summarized in Table 1. As seen in Figure 2a, all polymers exhibited absorption bands in the visible region, which are attributable to the ICT complex between donors units (aniline or fluorene) and acceptor moieties (quinoxaline or benzazole derivatives). Absorption bands due to π-π* bencenoid transitions were also observed between 275 and 375 nm.

Due to their strong electron-withdrawing nature, the absorption maxima of polymers containing benzothiadiazole moieties are red-shifted compared with those containing quinoxaline units. Besides, polymers with **TBQ** and **NTDz** portions are red-shifted related to **TQ** and **BTDz** moieties, respectively. This behavior was expectable due to their higher *M_w_* and the fused ring structure of the acceptor monomers, which extend the conjugation length of the molecular backbones [41]. The optical bandgaps (*E_g_^opt^*) estimated from the absorption edges are in the order of **PAFC6TQ** (2.54 eV), **PAFC6BTDz** (2.42 eV), **PAFC6TBQ** (2.35 eV) and **PAFC6NTDz** (2.16 eV). Although the absorption maximum of **PAFC6TBQ** is blue-shifted compared with **PAFC6BTDz**, the ICT band of **PAFC6TBQ** exhibits an extended tail towards low-energy wavelength, which decreases its bandgap value. The extended tail suggests stronger interactions between **PAFC6TBQ** chains, probably because the polymer tends to adopt a planar conformation driven by **TBQ** and **FC6** monomers.

Despite the decrease in their molecular weights, 2D-CPs tend to absorb slightly more towards low-energy wavelengths than 1D-CPs. The overlapping between the p-orbitals of the main chain with the p-orbitals of PANI segments, which increases the extension of conjugation, would partially explain these results. This effect could be probably more significant if the PANI growth were more regular and controlled. Further, no polaronic bands of PANI were observed in the region of 505–733 nm due to the polymers being reduced with hydrazine [42].

Figure 2b shows the fluorescence spectra of 1D- and 2D-conjugated copolymers. As seen, the same trend of UV-Vis spectra can be observed; polymers based on **NTDz** unit exhibit the most red-shifted emission bands (red region of the visible spectrum), while polymers based on **TQ** moieties present the most blue-shifted emission bands (yellow region of the visible spectrum). No significant differences between the emission maxima of 1D- and 2D-CPs were observed. However, fluorescence spectra without normalizing show a quenching process in 2D-CPs compared to statistical D-A CPs. The quenching phenomena reflect the effective electronic communication between the polymer backbone and its PANI branches.

**Figure 3:**
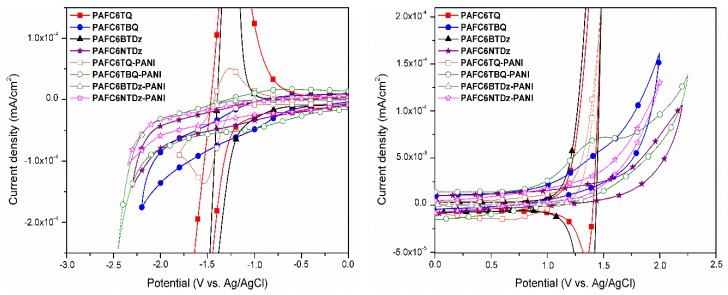
Cyclic voltammograms of 1D- and 2D-CPs deposited into fluorine-doped tin oxide (FTO).

Cyclic voltammetry studies were carried out to investigate the electrochemical redox properties of the polymers. As shown in Figure 3, all polymers exhibit one oxidation process and one reduction process. Except for **PAFC6TQ** and **PAFC6BTz**, all polymers present irreversible or low current density redox processes under the experimental conditions.

**Figure 4:**
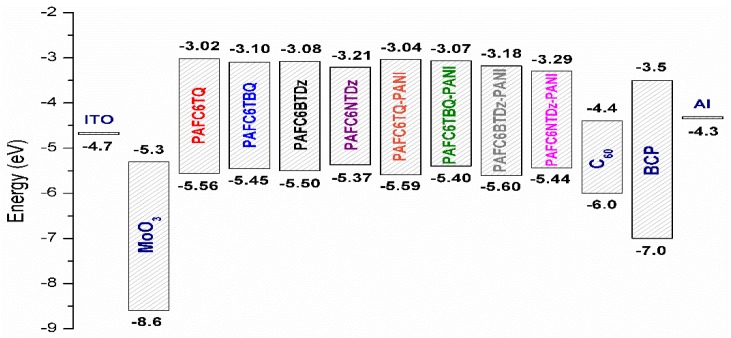
Device structure and HOMO and LUMO energy levels of 1D- and 2D-conjugated copolymers.

The HOMO energy levels of 1D- and 2D-CPs were estimated from the oxidation onset potentials *E_on_^ox^*, assuming the SCE electrode to be −4.8 eV from the vacuum, while the LUMO levels were calculated based on the difference between the HOMO level determined by cyclic voltammetry and the *E_g_^opt^* [43,44]. These results are summarized in Table 1 and represented in Figure 4. As shown in Table 1, the polymers present HOMO energy levels between −5.60 and −5.37 eV. The HOMO levels for 1D-CPs tend to increase in the order of **PAFC6NTDz** > **PAFC6TBQ** > **PAFC6BTDz** > **PAFC6TQ**. Since the donor moieties are the same in all polymers, this trend could be related to the nature of the acceptor units. Stronger electron-withdrawing groups in the acceptor monomers result in lower HOMO energy levels in the corresponding copolymer, as stated in previous studies [45]. It was expected that 2D-CPs would have lower HOMO values than 1D-CPs due to the ability of the PANI segments to stabilize radical cations or dications [46]; however, the results do not reflect this behavior clearly, probably due to the irregular growth of PANI segments onto the statistical D-A CPs.

On the other hand, the LUMO levels of 1D- and 2D-CPs ranged from −3.29–−3.02 eV. The LUMO values were lower for the polymers with **NTDz** and **TBQ** acceptor units in comparison with the **TQ**- and **BTDz**-based copolymers, which shows the effect exerted by the increment of the benzene ratio in the acceptor monomers. According to the energy diagram in Figure 4, the HOMO and LUMO of levels of the 1D- and 2D-CPs are well aligned with the HOMO and LUMO of MoO_3_ and fullerene (C_60_). These results are quite promising given that for an efficient device operation, it is necessary to have HOMO and LUMO levels of the emissive layer suitably aligned with the HOMO and LUMO of hole-transport and electron-transport materials.

### 3.3. Morphological Characterization

**Figure 5:**
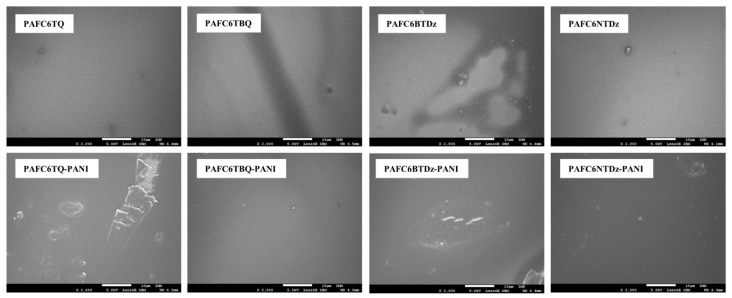
SEM images of 1D- and 2D-conjugated copolymers deposited on glass/ITO/MoO_3_ substrates. Scale bar: 10 μm.

To gain insight into the morphology of the 1D- and 2D-CPs, SEM microscopy was used to investigate the surface of their films. The polymers were deposited by dip coating onto glass/ITO/MoO_3_ substrates from a chloroform solution with a concentration of 3 mg/mL. The SEM images are given in Figure 5. As seen, relatively smooth surfaces are observed for **PAFC6TQ, PAFC6TBQ-PANI**, **PAFC6NTDz** and **PAFC6NTDz-PANI**, while the other polymers form less homogeneous deposits. It is known that interfacial morphology plays a crucial role in both charge collection and transport in opto-electronic devices and that better device performances can be obtained the more smoothed and homogeneous the surfaces are [15,47]. On the contrary, **PAFC6TQ-PANI** and **PAFC6BTDz-PANI** exhibit large surface defects, which could be attributed to their reduced ability to form films due to their low molecular weights. These latter results indicate non-optimal morphologies and will probably limit the efficiency of the resulting devices.

### 3.4. Electroluminescence Characteristics

**Figure 6:**
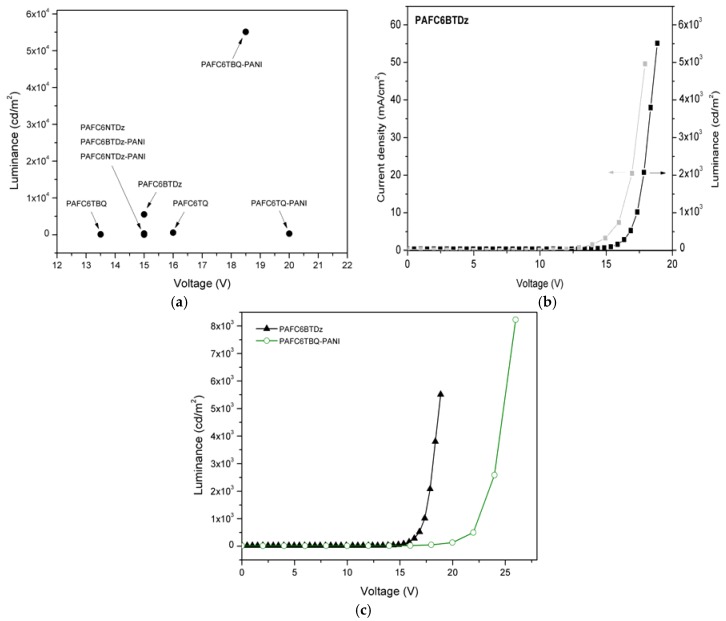
Device characteristics of the conducting polymer (CP)-based OLEDs with the configuration of glass/ITO/MoO_3_ (3 nm)/polymer (50–60 nm)/C_60_ (40 nm)/bathocuproine (BCP) (10 nm)/Al (100 nm). (**a**) Luminance-voltage values for the best performing devices with 1D- and 2D-CPs as emissive layers. (**b**) Current density-voltage-luminance plot of the **PAFC6BTDz**-based device. (**c**) Luminance-voltage characteristics of the **PAFC6BTDz** and **PAFC6TBQ-PANI** as emitting CPs.

OLED devices were fabricated using the 1D- and 2D-CPs as emissive layer materials. The solution-processed OLEDs were made with the configuration of glass/ITO/MoO_3_ (3 nm)/1D- or 2D-CP (50–60 nm)/C_60_ (40 nm)/BCP (10 nm)/Al (100 nm). The luminance-voltage properties for the best performing 1D- and 2D-CP-based devices are depicted in Figure 6a.

It must be noted that to obtain reproducible results, it is necessary to produce polymer films corresponding to five or six dip-coating cycles, i.e., films 50–60 nm thick. For a smaller number of cycles, the results are far less reproducible due to the appearance of leakage currents induced by the presence of small pinholes [48,49]. Obviously, the leakage current can be reduced by increasing the polymer layer thickness, as we tested. Nevertheless, the relatively high polymer layer thickness may justify, at least partially, the surprisingly high values of the turn-on voltages (V_on_) corresponding to the fast increase of the current and the electroluminescence apparition. Furthermore, differences of the V_on_ obtained from I-V characteristics and EL-V characteristics were observed. For example, the V_on_ estimated from the I-V curve of **PAFC6BTDz** is around 13.5 V, while the measured from the EL-V plot is about 15 eV (Figure 6b). As is known, the I-V characteristics are determined by the majority charge-carriers, while the EL-V characteristics are determined by the minority charge-carriers. Therefore, the difference between both V_on_ reflects the relative energy barrier height for the charge injection and the imbalance of the charges [49].

The V_on_ values of the 1D-CP series are around 15 V, while for the 2D-CP series, they depend on the polymer, with a general tendency towards higher values, which may result in an irreversible burning effect (Figure 6a). Apparently, the presence of PANI increases the threshold value, which was unexpected due to its well-known good electric conductivity. This could be related to its irregular growth onto 1D-CPs, which increases the morphological disorder of the films and, therefore, their resistivities. Besides, 1D-CP-based devices tend to exhibit higher luminance values than 2D-CP-based devices, except for **PAFC6TBQ-PANI**-based OLEDs, which present a surprisingly high luminescence of 55,100 cd/m^2^ and a current density of 65 mA/cm^2^, the highest values of the series. However, such high performance was achieved at 18.5 V, before the device became ruined. This promising luminescence result, along with that obtained for **PAFC6BTDz** (Figure 6c), deserves more specific studies in the future.

Despite the successful incorporation of PANI segments onto the 1D-CP backbones, the suitable energy levels of the materials and the good morphology of some films, the EL devices based on 2D-CPs presented low performances. From the values obtained from this exploratory research, in further studies, we will attempt to improve the device performances by controlling in a better way the thickness of the active layer and improving its film quality. Moreover, we also will focus on producing 2D-CPs with higher molecular weights and with regular PANI segments or blocks.

## 4. Conclusions

In summary, a new series of statistical D-A polymers (1D-CPs) based on aniline and 9,9-dihexylfluorene as donor moieties and benzo- or naphtho-quinoxaline/thiadiazole derivatives as acceptor units, presenting PANI segments as side chains (2D-CPs), was successfully synthesized. To assess the effects of electronic communication between the main chain and the side chains, absorption and fluorescence spectra, morphology characteristics and electrochemical and electroluminescent properties were measured. The principal results of this exploratory research indicate that some degree of PANI irregularly grew onto the main chains of 1D-CPs. As a consequence of the functionalization process, the 2D-CPs had lower molecular weights than statistical D-A polymers. UV-Vis and fluorescence spectra show that a partial overlap occurs among the p-orbitals of the main chain with the PANI segments and that 2D-CPs tend to absorb and emit light slightly red-shifted in comparison with 1D-CPs, attributable to the side-chains’ presence. These new 1D- and 2D-materials evidence emission bands between the yellow and red region of the visible spectra. CPs also exhibit optical bandgaps in the range of 2.15–2.55 eV, and fine-tuned HOMO (−5.37–−5.60 eV) and LUMO (−3.02–−3.29 eV) energy levels. Furthermore, the polymers with the highest molecular weights of the series tend to form smoother and more homogeneous surfaces, according to SEM images. Besides, OLED devices using CPs as emissive layers were fabricated. The preliminary results obtained show low performances of the devices, in which the electroluminescence originates at high values of the turn-on voltages. In the following studies, we will focus on producing 2D-CPs with higher molecular weights, with more regular segments of PANI, and on having better control of the emissive layer thicknesses, to produce OLEDs with better performances.

## Figures and Tables

**Table 1:** Physical properties of 1D- and 2D-conjugated polymers.

Polymer	*M_w_* (Kg/mol) ^a^	PDI ^a^	λ_max_^abs^ (nm) ^b^	λ_max_^em^ (nm) ^c^	*E_HOMO_* (eV) ^d^	*E_LUMO_* (eV) ^e^	*E_g_^opt^* (eV) ^f^
**PAFC6TQ**	4.8	1.58	407	516	−5.56	−3.02	2.54
**PAFC6TBQ**	14.8	2.37	423	570	−5.45	−3.10	2.35
**PAFC6BTDz**	6.3	1.57	442	529	−5.50	−3.08	2.42
**PAFC6NTDz**	67.8	2.65	503	603	−5.37	−3.21	2.16
**PAFC6TQ-PANI**	2.7	1.92	408	517	−5.59	−3.04	2.55
**PAFC6TBQ-PANI**	3.9	1.55	426	571	−5.40	−3.07	2.33
**PAFC6BTDz-PANI**	4.6	2.24	438	529	−5.60	−3.18	2.42
**PAFC6NTDz-PANI**	24.6	3.29	508	603	−5.44	−3.29	2.15

^a^ Weight-average molecular weight and polydispersity index determined by SEC. ^b^ Absorption maximum taken from UV-Vis spectra of the polymer in thin film. ^c^ Emission maximum taken from fluorescence spectra. ^d^ *E_HOMO_* = −e(*E_on_^ox^* + 4.4) eV. ^e^ *E_LUMO_* = (*E_HOMO_* + *E_g_^opt^*) eV. ^f^ Calculated from the absorption edge of the polymer films: *E_g_^opt^* = 1240/λ_edge_.

## References

[1] Holliday S., Li Y., Luscombe C.K. (2017). Recent advances in high performance donor-acceptor polymers for organic photovoltaics. Prog. Polym. Sci..

[2] Hu Z., Ying L., Huang F., Cao Y. (2017). Towards a bright future: Polymer solar cells with power conversion efficiencies over 10 %. Sci. China. Chem..

[3] Zhao W., Li S., Yao H., Zhang S., Zhang Y., Yang B., Hou J. (2017). Molecular optimization enables over 13% efficiency in organic solar cells. J. Am. Chem. Soc..

[4] Zhong W., Cui J., Fan B., Ying L., Wang Y., Wang X., Zhang G., Jiang X.-F., Huang F., Cao Y. (2017). Enhanced photovoltaic performance of ternary polymer solar cells by incorporation of a narrow-bandgap nonfullerene acceptor. Chem. Mater..

[5] Shin E.-S., Ha Y.H., Gann E., Lee Y.-J., Kwon S.-K., McNeill C.R., Noh Y.Y., Kim Y.-H. (2018). Design of new isoindigo based copolymer for ambipolar organic field-effect transistors. ACS Appl. Mater. Interfaces.

[6] Lim C.J., Li L., Lei Y., Zhou F., Wu B., Liu X., Zhu F., Ong B.S., Hu X., Su H. (2016). Synthesis and characterization of three thienopyridazine-based copolymers and their application in OFET. Tetrahedron Lett..

[7] Kim G., Kim H., Jang M., Jung Y.K., Oh J.H., Yang C. (2016). Ultra-narrow-bandgap thienoisoindigo polymers: Structure–property correlations in field-effect transistors. J. Mater. Chem. C.

[8] Wong M.Y. (2017). Recent advances in polymer organic light-emitting diodes (PLED) using non-conjugated polymers as the emitting layer and contrasting them with conjugated counterparts. J. Electron. Mater..

[9] Griniene R., Tavgeniene D., Baranauskyte U., Xie Z., Zhang B., Gelzinis A., Grigalevicius S. (2017). New electroactive polymers with electronically isolated 3,6,9-triarylcarbazole units as efficient hole transporting materials for organic light emitting diodes. Opt. Mater..

[10] Liang J., Zhao S., Jiang X.-F., Guo T., Yip H.-L., Ying L., Huang F., Yang W., Cao Y. (2016). White polymer light-emitting diodes based on exciplex electroluminescence from polymer blends and a single polymer. ACS Appl. Mater. Interfaces.

[11] Lombeck F., Di D., Yang L., Meraldi L., Athanasopoulos S., Credgington D., Sommer M., Friend R.H. (2016). PCDTBT: From Polymer Photovoltaics to Light-Emitting Diodes by Side-Chain-Controlled Luminescence. Macromolecules.

[12] Chen X.-Q., Yao X., Bai T., Ling J., Xiao W.-J., Wang J., Wu S.-C., Liu L.-N., Xie G., Li J. (2018). Donor-acceptor photovoltaic polymers based on 1,4-dithienyl-2,5-dialkoxybenzene with intramolecular noncovalent interactions. J. Polym. Sci. A Polym. Chem..

[13] Song H., Deng Y., Gao Y., Jiang Y., Tian H., Yan D., Geng Y., Wang F. (2017). Donor–acceptor conjugated polymers based on indacenodithiophene derivative bridged diketopyrrolopyrroles: Synthesis and semiconducting properties. Macromolecules.

[14] Yang Y., Wang J., Zhan X., Chen X. (2017). Designing a thiophene-fused benzoxadizole as an acceptor to build a narrow bandgap polymer for all-polymer solar cells. RSC Adv..

[15] Jessop I.A., Bustos M., Hidalgo D., Terraza C.A., Tundidor-Camba A., Pardo M.A., Fuentealba D., Hssein M., Bernede J.C. (2016). Synthesis of *2H*-benzotriazole based donor-acceptor polymers bearing carbazole derivative as pendant groups: Optical, electronical and photovoltaic properties. Int. J. Electrochem. Sci..

[16] Guo X., Baumgarten M., Müllen K. (2013). Designing ϖ-conjugated polymers for organic electronics. Prog. Polym. Sci..

[17] Höfle S., Zhang M., Dlugosch J., Kuhn M., Hamburger M., Colsmann A. (2017). Thermo-cleavable poly(fluorene-benzothiadiazole) to enable solution deposition of multi-layer organic light emitting diodes. Org. Electron..

[18] Wang J., Liu K., Ma L., Zhan X. (2016). Triarylamine: Versatile platform for organic, dye-sensitized, and perovskite solar cells. Chem. Rev..

[19] Wang P.-O., Shie W.-R., Jiang J.-C., Li L.-J., Liaw D.-J. (2016). Novel poly(triphenylamine-alt-fluorene) with asymmetric hexaphenylbenzene and pyrene moieties: Synthesis, fluorescence, flexible near-infrared electrochromic devices and theoretical investigation. Polym. Chem..

[20] Santos J., Cook J.H., Al-Attar H.A., Monkman A.P., Bryce M.R. (2015). Fluorene co-polymers with high efficiency deep-blue electroluminescence. J. Mater. Chem. C.

[21] Vásquez-Guilló R., Falco A., Martínez-Tomé M.J., Reyes Mateo C., Herrero M.A., Vázquez E., Mallavia R. (2018). Advantageous microwave-assisted Suzuki polycondensation for the synthesis of aniline-fluorene alternate copolymers as molecular model with solvent sensing properties. Polymers.

[22] Li F., Zou Y., Wang S., Fang L., Lutkenhaus J.L. (2017). Scalable synthesis and multi-electron transfer of aniline/fluorene copolymer for solution-processable battery cathodes. Macromol. Rapid Commun..

[23] Bin H., Gao L., Zhang Z.-G., Yang Y., Zhang Y., Zhang C., Chen S., Xue L., Yang C., Xiao M. (2016). 11.4% efficiency non-fullerene polymer solar cells with trialkylsilyl substituted 2D-conjugated polymer as donor. Nat. Commun..

[24] Bin H., Zhang Z.-G., Gao L., Chen S., Zhong L., Xue L., Yang C., Li Y. (2016). Non-fullerene polymer solar cells based on alkylthio and fluorine substituted 2D-conjugated polymers reach 9.5 % efficiency. J. Am. Chem. Soc..

[25] Zhang Z.-G., Li Y. (2015). Side-chain engineering of high-efficiency conjugated polymer photovoltaic materials. Sci. China Chem..

[26] Ye L., Zhang S., Huo L., Zhang M., Hou J. (2014). Molecular design toward highly efficient photovoltaic polymers based on two-dimensional conjugated benzodithiophene. Acc. Chem. Res..

[27] Choi M.-R., Woo S.-H., Han T.-H., Lim K.-G., Min S.-Y., Yun W.M., Kwon O.K., Park C.E., Kim K.-D., Shin H.-K. (2011). Polyaniline-based conducting polymer compositions with a high work function for hole-injection layers in organic light-emitting diodes: Formation of ohmic contacts. ChemSusChem.

[28] Bejbouji H., Vignau L., Miane J.L., Dang M.-T., Oualim E.M., Harmouchi M., Mouhsen A. (2010). Polyaniline as a hole injection layer on organic photovoltaic cells. Sol. Energy Mater. Sol. Cells.

[29] Kim J., Yun M.H., Kim G.-H., Kim J.Y., Yang C. (2012). Replacing 2,1,3-benzothiadiazole with 2,1,3-naphthothiadiazole in PCDTBT: Towards a low bandgap polymer with deep HOMO energy level. Polym. Chem..

[30] Balan A., Baran D., Toppare L. (2011). Benzotriazole containing conjugated polymers for multipurpose organic electronic applications. Polym. Chem..

[31] Tsubata Y., Suzuki T., Miyashi T., Yamashita Y. (1992). Single-component organic conductors based on neutral radicals containing the pyrazino-TCNQ skeleton. J. Org. Chem..

[32] Wei P., Duan L., Zhang D., Qiao J., Wang L., Wang R., Dong G., Qiu Y. (2008). A new type of light-emitting naphtho[2,3-*c*][1,2,5]thiadiazole derivatives: Synthesis, photophysical characterization and transporting properties. J. Mater. Chem..

[33] Islami M.R., Hassani Z. (2008). One-pot and efficient protocol for synthesis of quinoxaline derivatives. Arkivoc.

[34] East G.A., del Valle M.A. (2000). Easy-to-Make Ag/AgCl Reference Electrode. J. Chem. Ed..

[35] Yapi A.S., Toumi L., Lare Y., Soto G.M., Cattin L., Toubal K., Djafri A., Morsli M., Khelil A., Del Valle M.A., Bernède J.-C. (2010). On the influence of the exciton-blocking layer on the organic multilayer cells properties. Eur. Phys. J. Appl. Phys..

[36] Padma R., Guhanathan S. (2016). Synthesis, characterisation and antibacterial activity of 2,3-difurylquinoxalin-6-vinyl-benzaldehyde. Chem. Sin..

[37] Ahmed B., Yusuf Md. (2010). Synthesis of aromatic aldehyde imine derivatives of 2-thiobenzyl-1,3,4-thiadiazole and evaluation of their anticonvulsant activity. Indian J. Chem. Sec. B.

[38] Marani A.D., Entezami A.-A. (1994). New synthesis method of polythiophenes. Iran. J. Polym. Sci. Technol..

[39] Arasi A.Y., Jeyakumari J.J.L., Sundaresan B., Dhanalakshmi V., Anbarasan R. (2009). Th estructural properties of Poly(aniline)–Analysis via FTIR spectroscopy. Spectrochim. Acta A.

[40] Hu H., Cadenas J.L., Saniger J.M., Nair P.K. (1998). Electrically conducting polyaniline-poly(acrylic acid) blends. Polym. Int..

[41] Shen P., Bin H., Chen L., Zhang Z.-G., Li Y. (2015). Synthesis and photovoltaic properties of 4,9-dithien-2′-yl-2,1,3-naphthothiadiazole-based D-A copolymers. Polymer.

[42] Ramachandran R., Kathiravan R., Rani M., Kabilan S., Jeong Y.T. (2012). Synthesis and characterization of novel conducting 1,5-naphthalenediamine–aniline copolymer. Synth. Met..

[43] Najari A., Beaupré S., Allard N., Ouattara M., Pouliot J.-R., Charest P., Besner S., Simoneau M., Leclerc M. (2015). Thieno, furo, and selenopheno[3,4-*c*]pyrrole-4,6-dione copolymers: Air-processed polymer solar cells with power conversion efficiency up to 7.1%. Adv. Energy Mater..

[44] Zamora P.P., Camarada M.B., Jessop I.A., Díaz F.R., del Valle M.A., Cattin L., Louarn G., Bernede J.C. (2012). Synthesis, characterization, morphology and photovoltaic properties of aniline-tiophene based polymers. Int. J. Electrochem. Sci..

[45] Kim B.-G., Ma X., Chen C., Ie Y., Coir E.W., Hashemi H., Aso Y., Green P.F., Kieffer J., Kim J. (2013). Energy level modulation of HOMO, LUMO, and band-gap in conjugated polymers for organic photovoltaic applications. Adv. Funct. Mater..

[46] Ćirić-Marjanović G. (2013). Recent advances in polyaniline research: Polymerization, mechanisms, structural aspects, properties and applications. Synth. Met..

[47] Zhang S., Ye L., Hou J. (2016). Breaking the 10% efficiency barrier in organic photovoltaics: Morphology and device optimization of well-known PBDTTT polymers. Adv. Energy Mater..

[48] Brovelli F., Díaz F.R., del Valle M.A., Bernede J.C., Molinie P. (2001). Organic light-emitting devices of thiophene vynilic derivatives. Synth. Met..

[49] Ouro Djobo S., Bernède J.C. (2004). PVK/Alq_3_ organic light emitting diodes obtained by evaporation. J. Mater. Sci. Mater. Electron..

